# Herpes Zoster Without Vesicles: An Atypical Linear Erythematous Plaque on the Inner Thigh

**DOI:** 10.7759/cureus.81825

**Published:** 2025-04-07

**Authors:** Khadijah H Muzaffar, Abdullah Albadri

**Affiliations:** 1 General Practice, Batterjee Medical College, Jeddah, SAU; 2 Dermatology, King Fahad General Hospital, Jeddah, SAU

**Keywords:** dermatomes, derm path, herpes zoster (hz), skin disease/dermatology, varicella-zoster virus (vzv)

## Abstract

Herpes zoster (HZ), caused by the reactivation of the varicella-zoster virus (VZV), typically presents with a painful, vesicular rash along a dermatomal distribution. This case reports an unusual presentation in a 44-year-old female with hypothyroidism and urticaria, who presented with a solitary, linear erythematous plaque on the inner thigh, associated with burning pain. Initial topical treatments were ineffective, but prompt antiviral therapy with acyclovir led to complete resolution within six weeks. The case highlights the diagnostic challenges posed by atypical presentations of HZ and the importance of early diagnosis and treatment to prevent complications, such as postherpetic neuralgia. It also emphasizes the need to consider HZ in differential diagnoses even in the absence of typical vesicular eruptions to facilitate timely intervention and improved patient outcomes.

## Introduction

Herpes zoster (HZ), commonly known as shingles, is a viral infection caused by the reactivation of the latent varicella-zoster virus (VZV), which remains dormant in the ganglionic neurons derived from neural crest cells [1]. The VZV reactivates and travels along nerve fibers peripherally in conditions of immunosuppression, reduced cell-mediated immunity in the elderly, and mechanical or psychological stress [2].

This results in a secondary infection characterized by painful vesicular eruptions on an erythematous base in a dermatomal distribution [2]. It mostly erupts in one or two adjacent dermatomes that do not cross the midline [1]. Once the skin lesions have healed, the pain might last for months or even years [3]. This condition is referred to as postherpetic neuralgia (PHN) [3].

There are three dermatomes that are more commonly involved: thoracic (50%-60%), cervical (10%-20%), and trigeminal (10%-20%) [3,4]. Other, less commonly involved dermatomes are lumbar (5%-10%) and sacral (5%) [4]. Additionally, 1.7% to 2% of all HZ cases involve the maxillary and mandibular branches without the ophthalmic branch, which is a relatively uncommon occurrence [5].

People over 50 are most likely to develop HZ, and the risk increases with age [6]. It is more prevalent in individuals with compromised immune systems, such as those with cancer, HIV (human immunodeficiency virus), or AIDS (acquired immunodeficiency syndrome), or those on immunosuppressive medications [7]. Rarely, VZV reactivation can cause meningitis or encephalitis, with headache, nuchal rigidity, or altered mental status being the main symptoms [7].

In the absence of a characteristic rash, diagnosing HZ can be difficult [2]. Currently, HZ infection can be confirmed by detecting VZV DNA through polymerase chain reaction (PCR) testing. This can be performed on cerebrospinal fluid (CSF), peripheral blood mononuclear cells (PBMCs), or lesion samples. Additional diagnostic confirmation can be achieved by assessing for anti-VZV IgM and/or IgG antibodies in serum or CSF. Detection of VZV DNA in CSF is particularly useful in cases with suspected central nervous system involvement, such as VZV encephalitis or meningitis [2]. However, in cases where there is a high degree of clinical suspicion, treatment often needs to begin empirically, as immunoglobulins are detected only 60% of the time in an acute setting [2,7].

This case report aims to highlight the unique presentation of HZ and the importance of prompt diagnosis and treatment, which can hasten recovery and prevent complications.

## Case presentation

**Figure 1:**
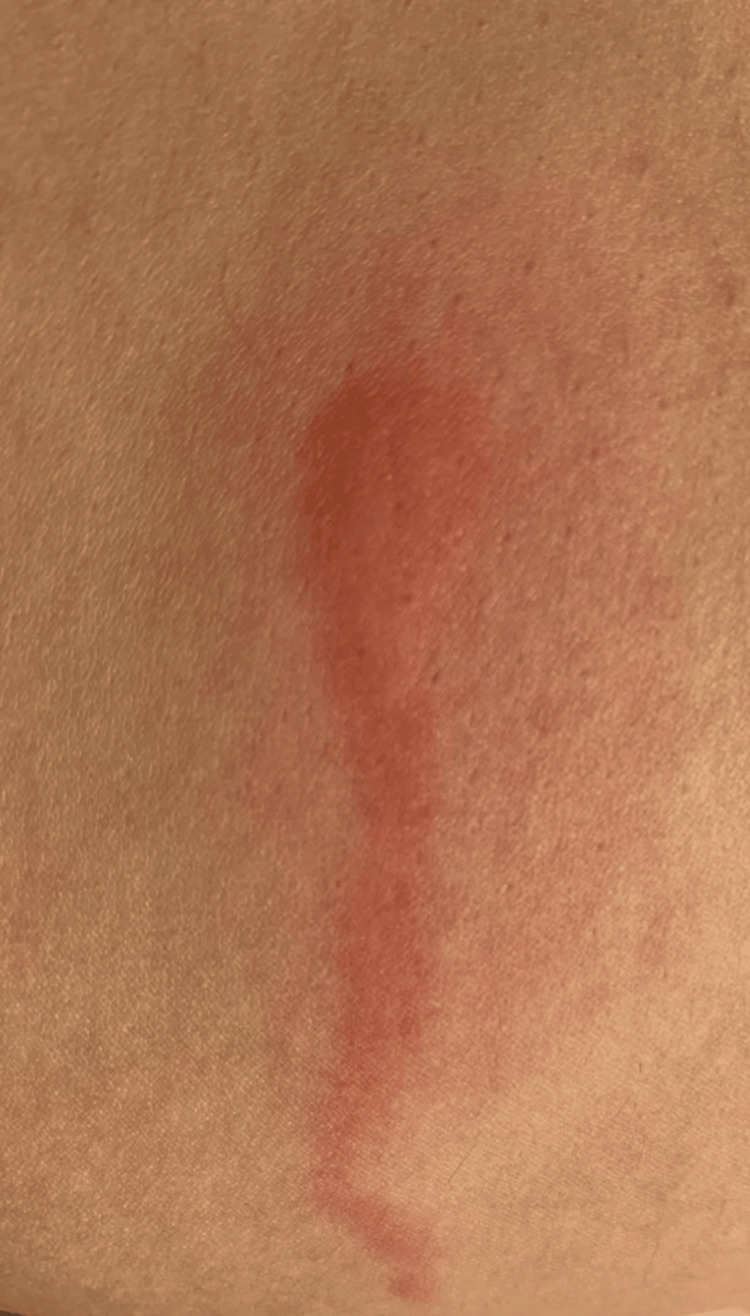
A linear erythematous and edematous plaque, on an erythematous background, following a dermatomal distribution on the inner left thigh

A 44-year-old female patient, known to have hypothyroidism and urticaria, presented with a history of redness in the inner left thigh for four days, associated with a burning sensation. It started suddenly with a single erythematous lesion, followed by pain with a burning sensation for three days and no itching (Figure 1).

Exacerbating factors included topical agents that the patient used at the beginning of her presentation; the burning sensation increased with tight pants and touch, with no relieving factors. She denied the use of immunosuppressants, acute illness, contact with sick patients, or emotional stress. Her previous treatments included mometasone furoate 0.1% for two days with no improvement, and Fucidin 2% for three days, which resulted in swelling and an increased burning sensation with both creams. Besides pain, the patient did not experience paresthesia, shock-like sensations, or dysesthesia. No other symptoms, such as headache, fever, general malaise, photophobia, night sweats, or weight loss, were noted. Other systemic reviews were unremarkable.

Regarding her past medical history, the patient has had hypothyroidism for 20 years and urticaria for one year. She does not recall having had a varicella-zoster infection. Her previous hospitalization or surgical intervention was due to a benign tumor in the lower jaw, diagnosed in 2004, treated with surgical excision, and without post-operative complications. She has no history of blood transfusion or any similar conditions in the past.

The patient’s current medications include levothyroxine 75 mcg once daily, montelukast 10 mg once daily, and cetirizine 10 mg twice daily. She does not have any allergies to food or medications. Family history is positive for diabetes and hypertension. The patient is a housewife, non-smoker, does not have pets at home, and has no recent travel history.

On examination, a solitary linear pinkish/red plaque on the left inner thigh was noted, warm on palpation. No investigations were done, and the first impression was HZ (shingles). The patient was started on acyclovir 800 mg PO, five times a day for seven days, Fucidin twice daily, and an antihistamine. After the first day of treatment, the patient’s burning sensation and swelling subsided, and improvement of erythema was noticed. Upon a six-week follow-up, the patient had complete resolution.

## Discussion

The lifetime risk of contracting HZ, an acute viral infection, is between 25% and 30% [1]. The incidence of HZ rises with age and in immunocompromised patients due to HIV or drug therapy [1,3]. It most often affects individuals over 45 years of age, with the highest incidence among those between 68 and 90 years [4]. In people 80 years of age and older, the risk rises to 50%, and in HIV-positive patients, it is 15 times higher than in non-infected individuals [1,3]. While those with reactivated infections can spread the VZV to non-immune contacts, HZ is not as contagious as the primary varicella infection [3,4].

HZ typically begins with a prodrome of mild-to-moderate burning or tingling in the skin of a particular dermatome, often associated with fever, headache, general malaise, and stomach upset [3]. Similar findings were reported in our cases, where the patient’s active issue was a burning sensation and pain but lacked systemic symptoms. A similar presentation, with no association to the dermatome but with symptoms of itching and burning, was seen in a case diagnosed first as balanitis but later confirmed as HZ after a positive VZV antibody IgM [1].

Several other atypical cases have been reported: Zhao et al. reported a case of disseminated HZ relapsing within one month despite antiviral and neurotrophic treatment [6]. Another recent study reported two cases of young, immunocompetent individuals: one presenting with sharp pain and a slightly raised, small erythematous lesion without vesicular changes, while the other presented with sharp burning pain without cutaneous manifestation, both resolving on antiviral therapy [2].

Our patient had a solitary, linear, erythematous lesion, different from the usual presentation of vesicular or maculopapular rash appearing along the dermatomes. Similar atypical reports include a skin eruption in the left lower limb with no history of chickenpox infection [8]. An atypical vesiculobullous eruption of HZ infection localized to the right foot and arm in a Saudi male with systemic lupus erythematosus [9]. Rare erythematous multidermatomal rashes have also been documented [10]. Interestingly, two recent studies reported a distinctive emergence of disseminated HZ in immunocompromised individuals [7,11].

The active stage of the disease is defined as the development of a unilateral erythematous, maculopapular rash along the dermatome within 48-72 hours of the prodrome [10]. This rash eventually transforms into a vesicular lesion [10]. Even the smallest stimuli can cause agonizing spasms due to the intensity of shingles pain, which ranges from mild to severe [2]. The diagnosis in our case was made clinically, based on the characteristic burning pain, dermatomal distribution, and rapid therapeutic response to acyclovir.

Other possible differential diagnoses, such as contact dermatitis, tinea corporis, or fixed drug eruption, were excluded based on the lesion’s unilateral, linear pattern, lack of itching, absence of scaling, and no history of new medication exposure. No investigations were performed, and empirical antiviral treatment was initiated early.

Early antiviral and symptomatic therapy initiation significantly lowers morbidity, since most virus replication stops 72 hours after the onset of rash; antiviral therapy should be started as soon as possible after cutaneous eruption [7,10]. Antivirals that are effective include guanosine analogs like valacyclovir (1000 mg, three times daily for seven days), famciclovir (500 mg, three times daily for seven days), and acyclovir (800 mg, five times daily for 7-10 days) [7]. Acyclovir decreases the intensity of acute pain, speeds up healing, stops the growth of new lesions, and shortens the time of viral shedding [1,10]. This is significant in our case, as the patient reported marked relief within 24 hours of starting treatment.

This case was not supported by laboratory confirmation, such as PCR testing or serologic assays for VZV. Routine laboratory investigations, including complete blood count (CBC) and inflammatory markers, were not conducted. This limits the strength of the case, especially given the atypical presentation. Future cases may benefit from appropriate diagnostic testing to confirm clinical suspicion and help exclude mimicking conditions.

It can be challenging to diagnose HZ when it manifests in unusual sites. Our article signifies the importance of early diagnosis. The exclusion of possible differential diagnoses, along with the consideration of burning pain as a striking symptom, can contribute to prompt diagnosis and prevent complications.

## Conclusions

This case demonstrates the importance of maintaining a high index of suspicion for HZ, even in the absence of classical symptoms such as vesicular eruptions. The patient’s presentation, which includes a solitary linear erythematous plaque with a burning sensation, without typical dermatomal vesicles, highlights the difficulties in diagnosing atypical HZ cases. Physicians should consider HZ as a differential diagnosis for unilateral painful skin lesions. For proper symptom resolution and prevention of complications, early clinical recognition and timely management with antiviral medication are necessary and critical, regardless of the presence or absence of vesicles, in reducing morbidity and enhancing patient outcomes.
